# Habituation of reflexive and motivated behavior in mice with deficient BK channel function

**DOI:** 10.3389/fnint.2013.00079

**Published:** 2013-11-19

**Authors:** Marei Typlt, Magdalena Mirkowski, Erin Azzopardi, Peter Ruth, Peter K. D. Pilz, Susanne Schmid

**Affiliations:** ^1^Department of Anatomy and Cell Biology, Schulich School of Medicine and Dentistry, University of Western OntarioLondon, ON, Canada; ^2^Pharmakologie and Toxikologie, Pharmazeutisches Institut, Universität TübingenTübingen, German; ^3^Tierphysiologie, Zoologisches Institut, Universität TübingenTübingen, German

**Keywords:** BK channel, sensorimotor gating, habituation, locomotion, startle

## Abstract

Habituation is considered the most basic form of learning. It describes the decrease of a behavioral response to a repeated non-threatening sensory stimulus and therefore provides an important sensory filtering mechanism. While some neuronal pathways mediating habituation are well described, underlying cellular/molecular mechanisms are not yet fully understood. In general, there is an agreement that short-term and long-term habituation are based on different mechanisms. Historically, a distinction has also been made between habituation of motivated versus reflexive behavior. In recent studies in invertebrates the large conductance voltage- and calcium-activated potassium (BK) channel has been implicated to be a key player in habituation by regulating synaptic transmission. Here, we tested mice deficient for the pore forming α-subunit of the BK channel for short-term and long-term habituation of the acoustic startle reflex (reflexive behavior) and of the exploratory locomotor behavior in the open field box (motivated behavior). Short-term habituation of startle was completely abolished in the BK knock-out mice, whereas neither long-term habituation of startle nor habituation of motivated behavior was affected by the BK deficiency. Our results support a highly preserved mechanism for short-term habituation of startle across species that is distinct from long-term habituation mechanisms. It also supports the notion that there are different mechanisms underlying habituation of motivated behavior versus reflexive behavior.

## INTRODUCTION

The brain constantly receives a vast amount of sensory information. In order to be able to extract salient information and respond appropriately, it is necessary to suppress repetitive non-informative input. One important sensory filtering mechanism responsible for suppression is habituation. Habituation describes the progressive decrease of a behavioral response to repetitive non-threatening sensory stimuli. It is considered to be the most basic form of learning and allows to ignore irrelevant stimuli in favor of relevant stimuli (Poon and Young, 2006). It is further believed to be a prerequisite for other learning forms (Rankin et al., 2009). In humans, disruption of habituation is strongly correlated with cognitive impairments. This was found in patients with mental disorders like schizophrenia (Geyer and Braff, 1982; Ludewig et al., 2003; Takahashi et al., 2008) and autism spectrum disorders (Ornitz et al., 1993; Perry et al., 2007).

While habituation is well characterized behaviorally (Thompson and Spencer, 1966; Groves and Thompson, 1970; Davis, 1984; Rankin et al., 2009), the underlying cellular/molecular mechanisms are not yet fully understood. Williams et al. (1974, 1975) suggested two different mechanisms of habituation by showing that habituation of motivated behavior was dependent on cholinergic mechanisms while the habituation of reflexive behavior was not. Also, the mechanisms for habituation within a session (short-term habituation) and between sessions (long-term habituation) seem to differ since they occur at different time scales and are largely independent from each other (Davis, 1984; Leaton et al., 1985).

Whereas mechanisms underlying habituation of motivated behavior have rarely been studied, habituation of reflexive behavior, especially of the startle response or similar escape responses, is relatively well studied in invertebrates and vertebrates, including humans. A calcium-dependent presynaptic depression mechanism in sensorimotor synaptic terminals within the primary startle pathway has been proposed to mediate short-term habituation of startle (Weber et al., 2002; Simons-Weidenmaier et al., 2006; Gover and Abrams, 2009). However, previous data have also shown that a common cause of synaptic depression, namely vesicle depletion, is unlikely to contribute substantially to the synaptic depression underlying habituation (Castellucci and Kandel, 1974; Byrne, 1982; Weber et al., 2002); the cause for synaptic depression therefore remains elusive. Recently, it has been shown that a loss-of-function mutation of the large conductance voltage- and calcium-activated potassium (BK) channel impairs short-term habituation of an escape response in *Drosophila* (Engel and Wu, 1998). BK channels are expressed throughout the mammalian nervous system (Knaus et al., 1996; Wanner et al., 1999; Sausbier et al., 2006) and can be found at neuronal soma and processes, as well as on presynaptic terminals (Knaus et al., 1996; Misonou et al., 2006; Sailer et al., 2006). They can be activated by membrane depolarization and micromolar concentrations of intracellular calcium and therefore can establish a link between intracellular free calcium, electrical excitability, and transmitter release in synaptic terminals (Robitaille et al., 1993; Yazejian et al., 1997; Sailer et al., 2006).

We here investigate the role of BK channels in both habituation of motivated and of reflexive behavior in mammals using mice that lack functional BK channels. We measured motor activity in a locomotor box, which reflects exploratory behavior in rodents (Crawley and paylor, 1997), in order to quantify motivated behavior. For measuring reflexive behavior, we used the acoustic startle response. We hypothesized that BK channel deficient mice show a disruption of short-term habituation of the startle response, corresponding to findings in *Drosophila* and *Caenorhabditis elegans*. We then tested whether BK channel knock-out mice also show disruptions in long-term habituation of startle. Finally, we analyzed whether BK channels play a role in habituation of motivated behavior, measuring exploratory behavior of BK channel knock-out mice in the locomotor box.

## MATERIALS AND METHODS

### ANIMALS AND ANIMAL CARE

We used mice of the F1 generation of a hybrid SV129/C57BL6 line with a deficient BK channel function bred at University of Tübingen. The BK channel function was abolished by deleting the *slo1* gene (accession ID# AAD49225.1) which encodes the pore forming channel protein (α-subunit; for details about generation of mice and genotyping please see supporting information in Sausbier et al., 2004). Heterozygous C57Bl6 mice were paired with heterozygous SV129 mice. Exclusively mice of the respective F1 generation, wild-type (WT), heterozygous (BKα^+^^/^^-^), and homozygous knock-out (BKα^-^^/^^-^), were tested in order to avoid effects of inbreeding. The animals were litter- and/or age matched. We only tested mice at ages from 3 to 5 months to avoid effects of aging.

All mice were generated and genotyped at the Pharmaceutical Institute, University of Tübingen, Germany. They were ear-tagged and shipped to Canada at the age of 1.5–3 months and subsequently quarantined and allowed to acclimate for 2 weeks before behavioral testing started. Mice were group housed with mixed genetic background within groups, with a 12 h light–dark cycle and with *ad libitum* food and water. Tails were marked according to their ear-tags for easy identification. Behavioral testing occurred during the light cycle. After all behavioral testing was finished, mice were sacrificed, and genotype was once more verified, comparing the ear-tags, shipping list, and tail marks.

All procedures were in accordance with the ethical guidelines of the Canadian Council on Animal Care (CCAC) and approved by the University of Western Ontario Animal Use Subcommittee.

### ACOUSTIC STARTLE REFLEX

**FIGURE 1:**
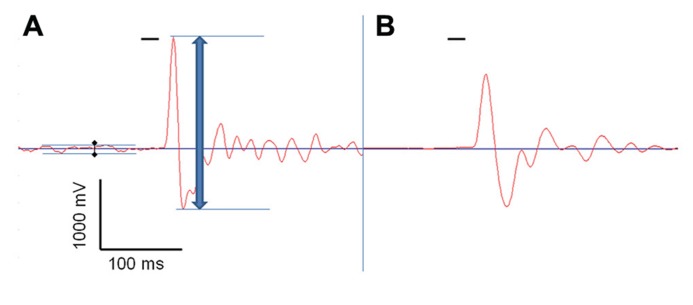
**Acoustic startle response measurement.** One acoustic startle response, measured by the Med Associates startle box, of a BK knock-out mouse (BKα^-^^/^^-^, **A**), and of a wild-type animal (WT, **B**), both recorded with a transducer signal gain of 1. The red line shows the voltage signal produced by the transducer that reflects the vertical dislocation of the platform by the animals’ movement, and the short horizontal black bar represents the startle stimulus. The blue arrow indicates the peak-to-peak amplitude measured by the system. The black diamonds indicate the baseline noise generated by the knock-out mouse’s tremor that was subtracted from the total startle amplitude.

Reflexive behavior was measured using the acoustic startle reflex. 18 WT (10 males/8 females), 17 BKα^+^^/^^-^ (10/7), and 19 BKα^-^^/^^-^ mice (9/10) were tested as described previously (Geyer and Swerdlow, 2001; Valsamis and Schmid, 2011). Testing was conducted in sound attenuated startle boxes from MED Associates (MED-ASR-PRO1, St Albans, VT, USA), where animals were placed into small encasements mounted on a movement sensitive platform within a sound attenuated chamber. A piezoelectric transducer mounted below the platform converted vertical movements of the platform induced by startle responses of the mouse into a voltage signal. The maximum amplitude (positive peak to negative peak) of the signal was measured in a 100 ms time window after the acoustic stimulus onset, using the associated software for stimulus presentation and recordings (see **Figure 1**; Startle Reflex version 6.0, MED Associates, Inc.).

Before the actual testing the animals were acclimatized to the startle boxes for 5 min on three consecutive days. During the acclimation periods only the background noise (65 dB sound pressure level, SPL, white noise) was presented. On the third day, acclimation was followed by a short input/output (I/O) test to determine the appropriate gain setting for amplifying the voltage signal of the transducer for each individual animal, so that a large portion of the dynamic range of the startle system was used for measuring startle in each animal: the I/O test consisted of 12 startle stimuli with increasing intensity starting at 65 dB SPL and increasing by 5 dB SPL each trial to 120 dB SPL (20 ms duration, white noise, every 20 s), presented on top of the background noise. The gain was set so that the maximum startle amplitude would cover a large portion of the dynamic range and the gain was subsequently kept constant for all recordings of a given animal. The absolute startle response amplitude was later corrected for the gain factor.

On the next five consecutive days the animals were tested using the following protocol: the animals were acclimatized to the startle box and the background noise for 5 min. Subsequently, the startle stimulus (20 ms, 105 dB SPL white noise) was presented 100 times with varying inter-trial intervals (10–20 s) on top of the background noise. Recordings started 50 ms before the stimulus was given and lasted for a total of 500 ms. To account for the muscular tremor occurring in the BKα^-^^/^^-^ mice, we subtracted the peak-to-peak transducer displacement during the phase before the startle pulse (50 ms) from the displacement measured during the startle pulse (see **Figure 1A**).

### OPEN FIELD LOCOMOTOR ACTIVITY

Motivated behavior was measured using open field locomotor activity that reflects exploratory behavior. Locomotor activity was measured in 16 mice of each genotype (WT: nine males/seven females, BKα^+^^/^^-^: 10/6, BKα^-^^/^^-^: 9/7). Each animal was placed in a squared (40 cm × 40 cm) open field box (Versamax animal activity monitor, AccuScan Instruments, Columbus, OH, USA) in a dimly lit room for five consecutive days and was allowed to explore freely for 2 h. In order to assess habituation, the distance traveled during these 2 h was analyzed in 5 min blocks using the VersaMax^TM^ software (AccuScan Instruments).

### DATA ANALYSIS AND STATISTICS

Data analyses and graphical display were done with Microsoft Excel (version 14.0.6129.5000, Microsoft Corp.), GraphPad (for graphic display only, Prism 6.01, GraphPad Software, La Jolla, CA, USA) and SPSS (for statistical analysis, version 20.0.0, IBM Corp.). Data for habituation curves are expressed as mean ± SE. For comparisons of baseline startle and habituation scores (see below), data is displayed as median and 25%/75% quartiles in box–whisker plots with whiskers indicating the 95th and 5th percentile.

In order to account for differences in the baseline, data of five subsequent trials were always averaged to a block value and these values were normalized to the first block within each day for assessing short-term habituation. For assessing long-term habituation, block one of each day was normalized to the first block on day 1. In order to compare the values across trials or days a repeated measurement ANOVA with genotype and gender as between-subjects factors was performed. We did not find a gender effect for any of the tested parameters, thus they were not reported separately in the Section “Results.” The Mauchly test was used to judge if the data violated the sphericity assumption. In case of a violation the degrees of freedom were corrected using the Greenhous–Geisser (if ∊ < 0.75) or the Huynh–Feldt method (if ∊ > 0.75).

In order to evaluate the amount of habituation we calculated a score for both, short- and long-term habituation. For short-term habituation the score is calculated as the ratio between the last block and the first block, whereas for long-term habituation it is the ratio between the first block of the last day of testing (day 5) and the first block of the first day of testing. A score of 1 indicates no habituation. The smaller the score, the more the animal habituated. A score > 1 indicates sensitization. The scores between genotypes were compared using a two-way-ANOVA (genotype × gender).

For *post hoc* analyses the Sidak *t*-test was performed to account for repetitive testing. Differences were considered statistically significant when *p*-values were smaller than 0.05. In the figures significance levels between genotypes were indicated as followed: *p ≤ 0.05, **p≤ 0.01, ***p ≤ 0.001.

## RESULTS

We tested the F1 generation of hybrid SV129/C57BL6 homozygous BKα knock-out mice (BKα^-^^/^^-^), as well as their heterozygous (BKα^+^^/^^-^), and WT littermates for short-term and long-term habituation of reflexive and motivated behavior.

### HABITUATION OF REFLEXIVE BEHAVIOR

**FIGURE 2:**
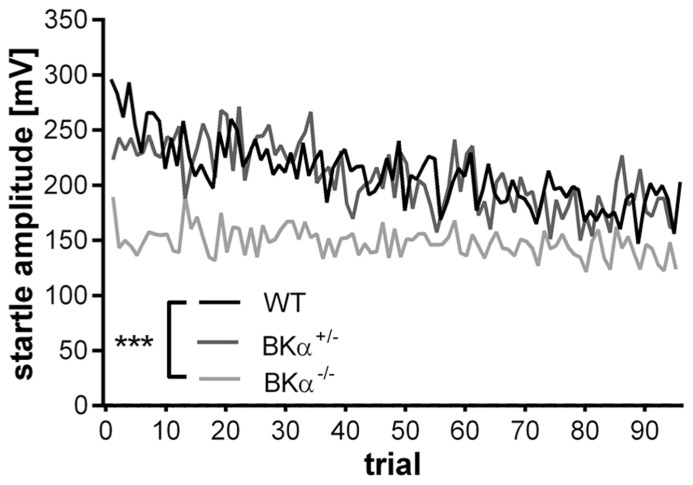
**Average startle response amplitudes of 100 trials.** Average startle response amplitudes of BKα^-^^/^^-^, BKα^+^^/^^-^, and WT mice. WT mice show a decline in response amplitude, whereas knock-out animals show no decline, with heterozygous animals intermediate. 18 WT (10 males/8 females), 17 BKα^+^^/^^-^ (10/7), and 19 BKα^-^^/^^-^ mice (9/10) were tested. ***p < 0.001.

**FIGURE 3:**
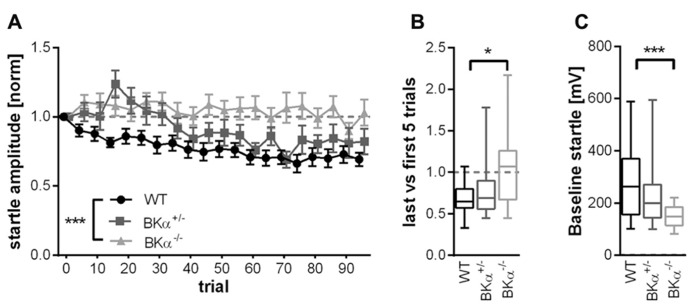
**Short-term habituation of the acoustic startle reflex.** **(A)** Of the 100 normalized startle response amplitudes of BKα^-^^/^^-^, BKα^+^^/^^-^, and WT mice, five consecutive startle responses were always averaged to one block. WT mice show a decline in response amplitude, whereas knock-out animals show no decline, with heterozygous animals intermediate. Error bars indicate standard error. ***p = 0.001. In **(B)** the habituation scores of the respective genotypes are displayed (first five versus last five responses, see Materials and Methods” for detailed description). Data is displayed as median in a box–whisker plot, with whiskers indicating the 5th and 95th percentile. *p = 0.009. **(C)** The baseline startle response was significant lower in knock-out animals than in WT. Data is displayed as median with whiskers indicating the 5th and 95th percentile. 18 WT (10 males/8 females), 17 BKα^+^^/^^-^ (10/7), and 19 BKα^-^^/^^-^ mice (9/10) were tested. ***p = 0.001.

The startle reflex to a sudden acoustic stimulus significantly decreased in the WT and BKα^+^^/^^-^ mice across testing trials within a session, but not in the BKα^-^^/^^-^ mice (**Figure 2**). A repeated measurement ANOVA (genotype × gender × trials) on amplitudes normalized to the first five trials (**Figure 3A**) reported a main effect for trial (*F*_(11.44,571.76__)_ = 5.58, *p* < 0.001), as well as a significant difference between genotypes (*F*_(2,50)_ = 8.57, *p* = 0.001) and an interaction between both (*F*_(22.87,571.75)_ = 1.74, *p* = 0.018). A *post hoc* test confirmed that habituation of the startle amplitudes in the BKα^-^^/^^-^ mice were significantly different from that of their WT littermates (*p* < 0.001).

In order to quantify the amount of habituation occurring, we calculated the short-term habituation scores as the ratio between the average of the last five and the first five trials. The ANOVA on these scores showed a significant effect for genotype (*F*_(2,50)_ = 5.10, *p* = 0.010). WT mice showed an average score of 0.70 ± 0.08 SE, which means they habituated on average by 30%. In contrast, the BKα^-^^/^^-^ mice did not habituate at all, with an average score of 1.03 ± 0.07 SE (*p* = 0.009). The scores of the heterozygote BKα^+^^/^^-^ mice fell in between the scores of WT and BKα^-^^/^^-^ mice and were not significantly different from either (*p* = 0.757, *p* = 0.119, respectively). **Figure 3B** shows the median habituation scores in a box–whisker plot in order to give an idea about the distribution of scores across animals.

Notably, the absolute startle amplitudes were significantly lower in the BKα^-^^/^^-^ mice (average 148 ± 9 mV SE) compared to their WT litter mates (273 ± 30 mV SE). A two-way ANOVA on the absolute startle amplitudes resulted in a significant genotype effect (*F*_(2,50)_ = 6.57, *p* = 0.003) and *post hoc* analysis showed that the difference was between the WT and the BKα^-^^/^^-^ mice (*p* = 0.001, see **Figure 3C**). Still, the startle amplitudes of the BKα^-^^/^^-^ mice were well above the noise level, which was at 43 ± 1 mV SE.

**FIGURE 4:**
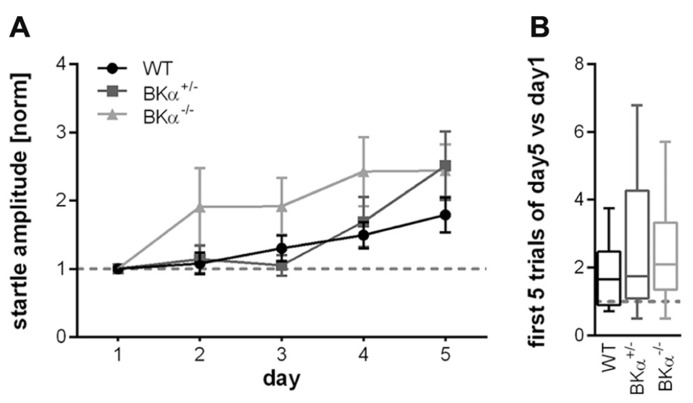
**Long-term habituation of the acoustic startle reflex.** **(A)** Normalized startle response amplitudes of BKα^-^^/^^-^, BKα^+^^/^^-^, and WT mice over five consecutive days. The amplitude of the first block (first five responses) per day were averaged for each day and normalized to the first day of each animal. None of the genotypes showed long-term habituation, but rather sensitization. There was no statistically significant difference between genotypes. Averaged data for genotypes and standard errors are displayed. In **(B)** the habituation scores (first five versus last five responses) of the respective genotypes are displayed as median and whiskers indicating the 5th and 95th percentile (see Materials and Methods for detailed description). 18 WT (10 males/8 females), 17 BKα^+^^/^^-^ (10/7), and 19 BKα^-^^/^^-^ mice (9/10) were tested.

Across 5 days of testing the initial startle amplitude increased in all animals suggesting that they sensitized to the stimulus between sessions rather than habituated. In order to account for the difference in baseline startle, amplitudes of the first block of each day were normalized to the first block of day 1 for each genotype. A repeated measurement ANOVA (genotype × gender × day) on normalized data reported an effect of day (*F*_(4,160)_ = 9.56, *p* < 0.001), but no significant effect of the genotype (*F*_(2,40)_ = 2.43, *p* = 0.101), nor a genotype × day interaction (*F*_(8,160)_ = 1.236, *p* = 0.281, **Figure 4A**). Accordingly, also the long-term habituation scores were not significantly different between genotypes (*F*_(2,40)_ = 0.785, *p* = 0.463, **Figure 4B**).

### HABITUATION OF MOTIVATED BEHAVIOR

**FIGURE 5:**
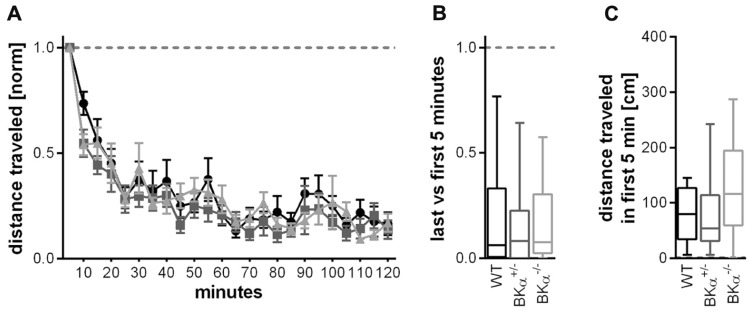
**Short-term habituation of open field locomotor activity.** **(A)** Habituation of exploratory behavior was measured in a locomotor box. Within the 2 h test in the open field box all animals habituated to the environment, leading to a strong decline in locomotion. Figure shows average values and standard errors. **(B)** All genotypes had a similar habituation score of around 0.2 (displayed as median and whiskers indicating the 5th and 95th percentile of first five versus last five time bins, see Materials and Methods). **(C)** Distance traveled (median) during the initial 5 min in the locomotor box for all three genotypes. There was no statistically significant difference in general locomotor activity between genotypes. 16 WT (nine males/seven females), 10 BKα^+^^/^^-^ (10/6), and 16 BKα^-^^/^^-^ (9/7) were tested.

Locomotor activity as a measure for exploratory behavior was assessed in a locomotor box (Crawley and paylor, 1997). Within the 2 h test in the open field box all animals habituated to the environment, leading to a strong decline in locomotion (**Figure 5A**). A repeated measurement ANOVA (genotype × gender × time) on the distance traveled within 5 min normalized to the distance traveled in the first 5 min showed a significant effect of time (*F*(_9.2_, _387.7_) = 31.45, *p* < 0.001), but no genotype effect (*F*_(2,42)_ = 1.50, *p* = 0.067), or a genotype × time interaction (*F*_(18.5,387.7)_ = 0.65, *p* = 0.860). In consequence, there was no significant difference between genotypes in the short-term habituation scores for the locomotor behavior (*F*_(2,42)_ = 0.848, *p* = 0.435), all genotypes had a similar score of around 0.2 (**Figure 5B**).

The absolute amount of locomotion was statistically not significantly different between genotypes. A two-way ANOVA on the absolute locomotion resulted in no significant genotype effect (*F*_(2,42)_ = 1.890, *p* = 0.164, **Figure 5C**).

**FIGURE 6:**
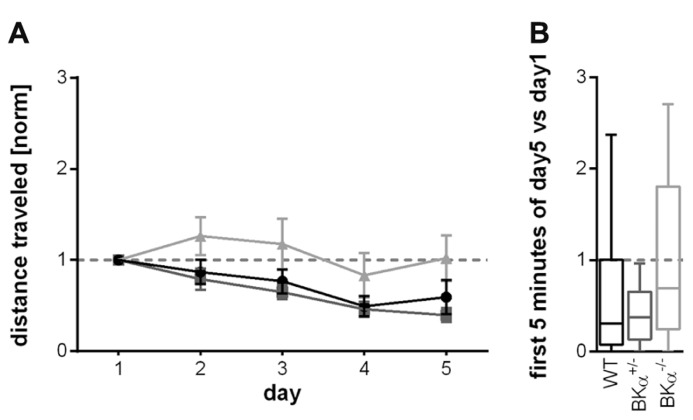
**Long-term habituation of open field locomotor activity.** **(A)** Changes in average locomotion across days showed no statistically significant difference between genotypes. Error bars indicate standard errors. **(B)** The long-term habituation scores (median, with whiskers indicating the 5th and 95th percentile) were also not significantly different between the genotypes. Knock-out animals show a slightly higher score (less long-term habituation), which is mainly due to an initial sensitization on day 2, followed by a decline.

Also the habituation across days did not significantly differ between genotypes for the locomotor behavior. The respective repeated measurement ANOVA (genotype × gender × day) only showed an effect for the day (*F*_(3.4,139.6)_ = 7.93, *p* < 0.001), but not for genotype (*F*_(2,41)_ = 2.72, *p* = 0.078). There also was no genotype × day interaction (*F*_(6.8,139.6)_ = 1.067, *p* = 0.39, **Figure 6A**). The long-term habituation scores were also statistically not significantly different between the genotypes (*F*_(2,41)_ = 2.41, *p* = 0.102, **Figure 6B**).

In summary, our study reveals and impact of deficient BK channel function on short-term habituation of the acoustic startle response, but not on short-term habituation of exploratory behavior, nor on long-term habituation.

## DISCUSSION

The present study shows a lack of short-term habituation of startle responses in BK channel knock-out mice, indicating a crucial role for BK channels in short-term habituation of reflexive responses, but no alterations in long-term habituation of startle or in habituation of exploratory behavior.

### LACK OF SHORT-TERM HABITUATION OF STARTLE IN BKα^-^^/^^-^ MICE

Our data clearly show that the BKα^-^^/^^-^ mice we used startled in response to a sound stimulus and that no habituation of this startle occurred. Several difficulties had to be considered in experimental design: it has been shown that a BK channel deficiency can alter locomotion (Sausbier et al., 2004) and hearing (Rüttiger et al., 2004; Oliver et al., 2006; Pyott et al., 2007; Kurt et al., 2012) which could affect the acoustic startle measures (as well as exploratory behavior in the locomotor box). We therefore used a F1 hybrid mouse in present study that has no hearing impairment in the relevant frequency spectrum (Typlt et al., 2013). Still, BKα^-^^/^^-^ mice showed a lower baseline startle response than their WT siblings. It is difficult to determine if this is due to lower body weight in BKα^-^^/^^-^ mice, lower anxiety levels, or motor impairments. We accounted for the lower baseline startle amplitude by normalizing the data of each mouse to its startle amplitude in the first trials. However, lower startle responses in general may influence the amount of habituation. The major concern is a floor effect, i.e., that startle response amplitude may not be sufficiently different from a general noise level and can therefore not be further reduced. However, the noise level in our experiments was still considerably lower than startle amplitudes of BKα^-^^/^^-^ mice. Furthermore, we subtracted the noise caused by tremor in knock-out mice so that it does not influence habituation scores. It also has been shown in the same BKα^-^^/^^-^ mice that startle responses are substantially reduced by prepulse inhibition (Typlt et al., 2013), indicating that there is still enough room for a substantial reduction of startle, which makes it unlikely that a floor effect accounts for the lack of habituation. We also looked at habituation exclusively in WT low startler with a baseline startle response comparable to BKα^-^^/^^-^. Although the number of WT low startler is too small to statistically analyze the data (*n* = 5), they all have habituation scores well below 1 (data not shown). We are therefore confident that there is a true lack of habituation in BKα^-^^/^^-^ mice.

### POSSIBLE ROLE OF THE BK CHANNELS IN SHORT-TERM HABITUATION OF STARTLE

It has been proposed that a calcium-dependent presynaptic depression mechanism in sensorimotor synaptic terminals within the primary startle pathway mediate short-term habituation of startle (Weber et al., 2002; Simons-Weidenmaier et al., 2006; Gover and Abrams, 2009). BK channels that are activated by depolarization and intracellular calcium drive the membrane potential towards the potassium equilibrium potential and therefore re- and hyperpolarize the neuron (Kaczorowski et al., 1996; Vergara et al., 1998; Poolos and Johnston, 1999; Hu et al., 2001). By limiting the duration of action potentials, they regulate the general excitability of neurons (Shao et al., 1999; Nelson et al., 2003; Brenner et al., 2005), as well as limit the transmitter release at presynaptic terminals (Robitaille and Charlton, 1992; Robitaille et al., 1993; Hu et al., 2001; Raffaelli et al., 2004; Wang, 2008). They co-localize with voltage-gated calcium channels at the active synaptic zone, establishing a link between intracellular free calcium and neurotransmitter release in synaptic terminals (Robitaille et al., 1993; Yazejian et al., 1997; Sailer et al., 2006). All these described properties make BK channels excellent candidates for mediating calcium-dependent synaptic depression in the startle pathway thereby causing habituation to repetitive strong stimuli.

Short-term habituation lasts for several minutes, whereas intracellular calcium is elevated in synaptic terminals only for milliseconds. So, how is synaptic depression maintained for minutes? BK channels can be phosphorylated by PKA, PKC, and most importantly by Ca^2^^+^/calmodulin dependent protein kinase II (CaMKII; Kaczorowski et al., 1996; Liu et al., 2007; Wang, 2008). The latter has been shown to be enriched in presynaptic terminals (Gorelick et al., 1988; Walaas et al., 1989) and to act as a strong regulator of synaptic strength and plasticity (Wang, 2008). CaMKII is activated by elevations of intracellular calcium and can auto-phosphorylate upon large calcium accumulation. Auto-phosphorylation leads to a prolonged activity of CaMKII that persists after calcium levels have returned to baseline. The prolonged activity of CaMKII leads to a prolonged phosphorylation of BK channels and therefore to a lasting decrease in synaptic efficacy (Wang, 2008). In fact, this proposed mechanism meets all requirements for a habituation mechanism, such as a presynaptic localization, calcium dependence, and the reversibility of the phosphorylation process within a timescale of several minutes.

Short-term habituation of startle in rodents has been shown to be mediated at the sensorimotor synapse in the pontine reticular formation where synaptic depression occurs upon repeated strong stimulation (Davis et al., 1982; Lingenhöhl and Friauf, 1992, 1994; Weber et al., 2002; Simons-Weidenmaier et al., 2006). Since the phosphorylation of BK channels requires a strong activation as described above, they are likely to mediate synaptic depression at this synapse, however, future electrophysiological experiments have to confirm this. It will also be intriguing to see in the future to what extend habituation of other reflexive behaviors depend on BK channel activation.

### POTENTIAL ROLE OF THE BK CHANNELS IN HABITUATION OF MOTIVATED BEHAVIOR

BK channels can potentially control transmitter release at any kind of synapse regardless of the type of neurotransmitter released. Furthermore, BK channels are expressed throughout the nervous system, so it could be hypothesized that their activation represents a universal mechanism for habituation. In our study, however, we found an effect of a functional BK channel deficiency only for short-term habituation of startle and not for long-term habituation nor habituation of motivated behavior. In fact, habituation of motivated behavior has been previously suggested to be based on separate mechanisms to habituation of reflexive behavior (Williams et al., 1974, 1975; Brown, 1976). Moreover, the proposed BK channel mechanism is unlikely to be able to mediate motivated behavior since in contrast to reflexive behavior there is no strong eliciting input for motivated behavior which could trigger the phosphorylation of CaMKII. Thus, a different mechanism is likely to account for habituation of motivated behavior.

### POTENTIAL ROLE OF THE BK CHANNELS IN LONG-TERM HABITUATION

Long-term habituation of startle has been shown to be located extrinsically to the primary startle pathway and involves the cortex and the cerebellar vermis (Leaton and Supple, 1986, 1991; Lopiano et al., 1990) and potentially cholinergic mechanisms (Schmid et al., 2011). It has been hypothesized to be an associative learning process. Since associative learning is affected by a lack of BK channel function (Matthews and Disterhoft, 2009; Typlt et al., 2013) we would have expected to see an effect of the BK channel deficiency on long-term habituation of startle as well. Unfortunately, neither WT- nor BK-deficient animals really showed long-term habituation of startle, which is common for many mouse strains. This makes it difficult to assess any differences between genotypes. The lack of statistically significant differences between genotypes in long-term habituation testing of both startle and locomotor behavior does therefore not completely rule out that there is a potential contribution of BK channels, for instance through their impact on associative learning

## CONCLUSION

The results of this study show that BK channel activation is necessary for short-term habituation of startle. It demonstrates that this mechanism underlying short-term habituation is highly preserved throughout evolution, since BK channel-dependent short-term habituation of a startle-like response has been found in *C. elegans* (C. Rankin, personal correspondence) and *Drosophila* (Engel and Wu, 1998). Additionally, genetic alterations of BK channel function has been implicated in different disorders in humans that are associated with short-term habituation deficits in startle, e.g., in schizophrenia (for review see Zhang et al., 2006), mental retardation (Higgins, 2008; Deng et al., 2013), and autism (Laumonnier et al., 2006). Furthermore, the fragile-x related protein, which is impacted in a specific form of autism in humans that is associated with a disruption of habituation has recently shown in mice to directly regulate BK channel activity (Deng et al., 2013). This highly preserved function of BK channels in habituation goes well with the notion of the importance of intact habituation for sensory filtering and higher cognitive function.

BK channels do not seem to play a role in short-term habituation of motivated behavior, and we could not find any evidence for a role in long-term habituation. This supports the idea that there is no universal habituation mechanism, but probably a variety of different mechanisms mediating habituation of different behaviors and at different time scales, as previously proposed (Williams et al., 1974, 1975; Davis, 1984; Leaton et al., 1985; Weber et al., 2002; Schmid et al., 2010).

## Conflict of Interest Statement

The authors declare that the research was conducted in the absence of any commercial or financial relationships that could be construed as a potential conflict of interest.

## AUTHOR CONTRIBUTIONS

Susanne Schmid conceptualized the study, consulting with Peter Ruth and Peter Pilz. Peter Ruth created the knock-out mice. Peter Pilz, Marei Typlt, and Magdalena Mirkowsi conducted experiments and analyzed data. Marei Typlt ran the stats, wrote the first draft of the manuscript, and made the figures. All authors revised the manuscript. Susanne Schmid finalized the manuscript and submitted it. Marei Typlt and Magdalena Mirkowski have contributed equally to the work.
